# Estimates of carbon stored in harvested wood products from the United States forest service northern region, 1906-2010

**DOI:** 10.1186/1750-0680-7-1

**Published:** 2012-01-13

**Authors:** Keith D Stockmann, Nathaniel M Anderson, Kenneth E Skog, Sean P Healey, Dan R Loeffler, Greg Jones, James F Morrison

**Affiliations:** 1 Northern Region, USDA Forest Service, Missoula, MT, USA; 2 Rocky Mountain Research Station, USDA Forest Service, Missoula, MT, USA; 3 Forest Products Laboratory, USDA Forest Service, Madison, WI, USA; 4 Rocky Mountain Research Station, USDA Forest Service, Ogden, UT, USA; 5 College of Forestry and Conservation, University of Montana, Missoula, MT, USA

## Abstract

**Background:**

Global forests capture and store significant amounts of CO_2 _through photosynthesis. When carbon is removed from forests through harvest, a portion of the harvested carbon is stored in wood products, often for many decades. The United States Forest Service (USFS) and other agencies are interested in accurately accounting for carbon flux associated with harvested wood products (HWP) to meet greenhouse gas monitoring commitments and climate change adaptation and mitigation objectives. This paper uses the Intergovernmental Panel on Climate Change (IPCC) production accounting approach and the California Forest Project Protocol (CFPP) to estimate HWP carbon storage from 1906 to 2010 for the USFS Northern Region, which includes forests in northern Idaho, Montana, South Dakota, and eastern Washington.

**Results:**

Based on the IPCC approach, carbon stocks in the HWP pool were increasing at one million megagrams of carbon (MgC) per year in the mid 1960s, with peak cumulative storage of 28 million MgC occurring in 1995. Net positive flux into the HWP pool over this period is primarily attributable to high harvest levels in the mid twentieth century. Harvest levels declined after 1970, resulting in less carbon entering the HWP pool. Since 1995, emissions from HWP at solid waste disposal sites have exceeded additions from harvesting, resulting in a decline in the total amount of carbon stored in the HWP pool. The CFPP approach shows a similar trend, with 100-year average carbon storage for each annual Northern Region harvest peaking in 1969 at 937,900 MgC, and fluctuating between 84,000 and 150,000 MgC over the last decade.

**Conclusions:**

The Northern Region HWP pool is now in a period of negative net annual stock change because the decay of products harvested between 1906 and 2010 exceeds additions of carbon to the HWP pool through harvest. However, total forest carbon includes both HWP and ecosystem carbon, which may have increased over the study period. Though our emphasis is on the Northern Region, we provide a framework by which the IPCC and CFPP methods can be applied broadly at sub-national scales to other regions, land management units, or firms.

## Background

Recent estimates of net annual storage, or flux, indicate that the world's forests are an important carbon sink, removing more carbon from the atmosphere through photosynthesis than they emit through combustion and decay [1]. The forest sector of the United States (US) stored about 48,437 teragrams of carbon (TgC) in 2010 [2], or the equivalent of about 30 years of US fossil fuel emissions at the 2008 rate. The US Environmental Protection Agency (EPA) estimates that in 2010 net additions to ecosystem and harvested wood products (HWP) pools were 235 TgC yr^-1 ^[2]. Thus, US forests function as a carbon sink, annually offsetting about 15 percent of the country's carbon emissions from fossil fuel combustion.

About 5 percent of total US forest sector carbon stocks and 6 percent of the annual flux is attributable to carbon in HWP [2]. Though the HWP fraction of the pool is small compared to ecosystem carbon, it is an important component of national level carbon accounting and reporting. As defined by the Intergovernmental Panel on Climate Change (IPCC), HWP are products made from wood including lumber, panels, paper, paperboard, and wood used for fuel [3]. The HWP carbon pool includes both products in use and products that have been discarded to solid waste disposal sites (SWDS). Additions to the HWP pool are made though harvesting and emissions are from decay and combustion of wood products.

Increasing social and managerial interest in mitigating rising atmospheric CO_2 _concentrations and the resulting impacts on climate has focused attention on the ecosystem service of forest carbon storage, including storage in HWP. Forest management can affect the quantity of carbon stored in both ecosystems and forest products over time, and management activities in the US frequently include silvicultural treatments that produce HWP. Credible information on forest ecosystem and HWP carbon stocks and fluxes can inform forest managers and the public of the tradeoffs between carbon storage and other forest management objectives, and between short and long-term carbon consequences of alternative forest management strategies [4-6].

As governments contemplate climate change mitigation and adaptation options, there is growing interest among forest managers in monitoring and managing forests for sequestration of carbon as an ecosystem service. For example, during 2010, the US Forest Service (USFS) developed a climate change scorecard that is to be completed annually for each of the 155 national forests and national grasslands managed by the agency [7]. The scorecard includes four categories of scored elements: organizational capacity; engagement; adaptation; and, mitigation and sustainable consumption. Elements under mitigation and sustainable consumption direct individual forests to develop a baseline assessment of carbon stocks, as well as an assessment of the influence of disturbance and management activities on these stocks. These assessments are meant to guide adaptation actions and continued monitoring. Managers are explicitly expected to begin integrating carbon stewardship with management of their forest for traditional multiple uses and other ecosystem services. These requirements necessitate robust and accessible monitoring systems that provide quantitative metrics to gauge progress. Policies and guidelines are currently under development regarding the appropriate level of accuracy needed for completing carbon assessments and for informing forest management decisions at the individual national forest level.

HWP carbon monitoring systems have been implemented at the national level [2,3,8], as well as at the level of an individual harvest [9]. Robust inventory-based methods for estimating carbon stocks and flux in forest ecosystems are well established in the US, with several tools available to forest managers [9-12]. However, many of the tools used to estimate carbon stored in forests, such as the Carbon On Line Estimator Version 2.0 [13] and the U.S. Forest Carbon Calculation Tool [14], do not provide estimates of HWP carbon and other tools are restricted to national level HWP accounting (e.g., WOODCARB II [3]). While these tools are relevant for public and industrial timber producers interested in documenting the carbon fluxes associated with harvesting activities [15], at their current scales of analysis they do not serve the needs of these forest managers. Managers need similarly accessible and practical tools for estimating and monitoring carbon stocks and flux in HWP at the agency or firm level [16].

### Objectives

There is a clear need for the means to monitor the contribution of HWP to carbon pools and greenhouse gas mitigation at sub-national scales. Currently, forest managers do not have the tools they need to accomplish monitoring goals that have been established at the national level. Developing these tools is an important step in facilitating carbon assessment and stewardship and in informing management actions on the ground. Our objectives with this study are to: 1) use established accounting approaches to make estimates of HWP carbon stocks and fluxes for the USFS Northern Region as a demonstration of sub-national HWP accounting, and 2) provide a framework with clear metrics and estimation methods that can be applied to other regions, land management units, or firms. We also provide guidance to managers concerning the differences between alternative approaches with regards to data and resource requirements. We do not develop a system for evaluating the future impacts of specific management actions, nor do we advocate any particular course of action to improve carbon stewardship.

### Accounting Approaches

**Figure 1:**
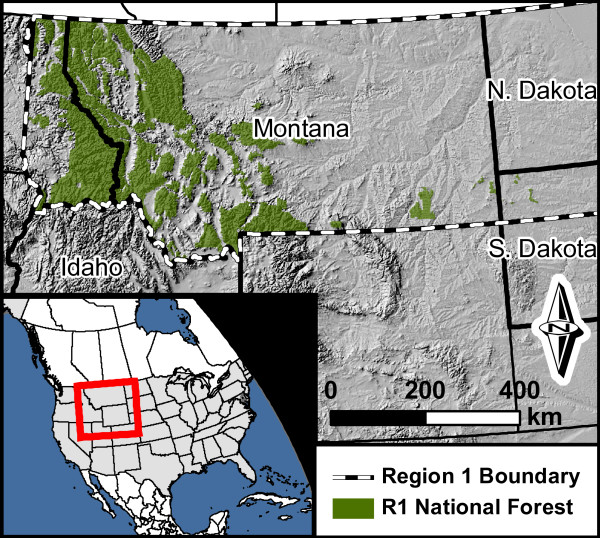
**Map of the study area**. The US Forest Service Northern Region (the Northern Region) administers approximately 8.1 million hectares of federally-owned forestland in the states of Montana, Idaho, South Dakota, and northeastern Washington.

We use the IPCC production accounting approach, which has been adopted by the US EPA; hereafter referred to as the IPCC/EPA approach), and the California Forest Project Protocol (CFPP; [17]) to estimate annual changes in HWP pools from 1906 to 2010 for the USFS Northern Region (Figure 1). The Northern Region contains approximately 10.9 million hectares of federally-owned land in the states of Montana, Idaho, North Dakota, South Dakota, and eastern Washington. Approximately 8.1 million hectares of this land are forested. We chose this region because it represents a management unit of the desired sub-national scale and managers in this region are particularly interested in developing tools to meet carbon stewardship objectives.

**Figure 2:**
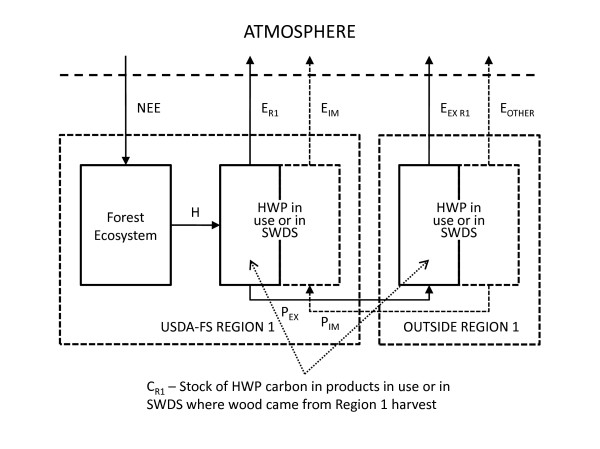
**Carbon flows and stocks associated with forest ecosytems and harvested wood products (HWP)**. Carbon flows and stocks associated with forest ecosystems and harvested wood products (HWP) are used to illustrate the IPCC/EPA production accounting approach (adapted from [3]).

In the IPCC/EPA production accounting approach, the annual carbon stock change for the total forest sector (ΔS) is a function of carbon flow among the atmosphere, forest ecosystems, and HWP, and is calculated as:

$$\Delta S=\;\left(NEE-H\right)\;+\;\left(\Delta C_{R1}\right)$$

where NEE is the annual net ecosystem exchange between the atmosphere and Northern Region forests from all ecosystem processes including photosynthesis, decay, and natural and anthropogenic fire, H is the annual harvest of wood from Northern Region forests for products, and ΔC_R1 _is the annual change in carbon stored in HWP that were made from wood harvested from Northern Region forests (Table 1, Figure 2). As discussed previously, the HWP pool is a relatively small but important fraction of the total forest sector carbon flux in the US, accounting for about 6 percent of the annual carbon stock change of 235 TgC yr^-1^.

**Table 1:** Variable definitions for the IPCC/EPA production accounting approach shown in Figure 2 [3]. Units for all variables are MgC yr^-1^.

Variable	Definition
ΔS	Annual carbon stock change for the total forest sector, which is calculated as Δ*S *= (*NEE*-*H*)+(Δ*C_R1_*) in the production accounting approach.
NEE	Annual net ecosystem carbon exchange, the annual net carbon that moves from the atmosphere to forests.
H	Annual harvest of wood for products, which includes wood and residues removed from harvest sites, but excludes resides left at harvest sites.
HWP	Harvested wood products in use or at solid waste disposal sites.
E_R1_	Annual emission of carbon to the atmosphere in the Northern Region from products made from wood harvested in the Northern Region.
E_IM_	Annual emission of carbon to the atmosphere in the Northern Region from products made from wood harvested outside of the Northern Region and imported into The Northern Region.
P_EX_	Annual exports of wood and paper products out of the Northern Region, including roundwood, chips, residue, pulp and recovered (recycled) products.
P_IM_	Annual imports of wood and paper products into the Northern Region, including roundwood, chips, residue, pulp and recovered (recycled) products.
E_EX R1_	Annual emission of carbon to the atmosphere in areas outside of the Northern Region from products made from wood harvested in the Northern Region.
E_OTHER_	Annual emission of carbon to the atmosphere in areas outside of the Northern Region from products made from wood harvested outside the Northern Region.
C_R1_	Stock of harvested wood products carbon in use or at solid waste disposal sites where products used wood from the Northern Region harvests.
ΔC_IU R1_	Annual change in carbon stored in harvested wood products in products in use where products used wood from the Northern Region harvests.
ΔC_SWDS R1_	Annual change in carbon stored in harvested wood products at solid waste disposal sites where products used wood from the Northern Region harvests.
ΔC_R1_	Annual change in carbon stored in harvested wood products in products in use and at solid waste disposal sites where products used wood from the Northern Region harvests. ΔC_R1 _= ΔC_IU R1 _+ ΔC_SWDS R1_

In this approach, the annual change in carbon stored in HWP (ΔC_R1_) is the sum of the net change in carbon stored in products in use (ΔC_IU R1_) and the net change in carbon stored in products at solid waste disposal sites (ΔC_SWDS R1_). Figure 2 shows that carbon emissions attributed to HWP from the Northern Region (indicated with solid boxes) include both emissions to the atmosphere from the Northern Region products that were used within the region (E_R1_) and emissions to the atmosphere from wood products harvested in the Northern Region that were exported outside the region (E_EX R1_). Exports (P_EX_) include wood and paper products, as well as roundwood, chips, residue, pulp and recovered (recycled) products from wood harvested in the Northern Region. Under the production accounting approach, imports from other regions (indicated with dotted lines around the right side portions for both boxes showing HWP in use or in SWDS are not included in Northern Region accounting because the emphasis is on the location of harvest (H). Emissions are further categorized as emitted with energy capture (e.g. fuelwood) and emitted without energy capture (e.g. decomposition and burning for waste disposal). The relevant metric for this accounting approach is annual stock change in the HWP carbon pool.

The CFPP was designed for application to smaller geographic areas and uses a simpler accounting approach focused on carbon storage for a single harvest year rather than net annual carbon change due to current year additions to product pools and current year emissions from those pools. The relevant metric for the CFPP accounting approach is 100-year average carbon stored from the current year's harvest - "the 100-year average." Like the production approach, the CFPP approach is applied to a specified area of land, and includes carbon stored in both products in use and SWDS. The approach uses mill efficiency factors and decay curves for individual product classes to estimate the average amount of carbon that is likely to remain stored in wood products from a given year's harvest over a 100-year period [9,18]. Specific estimation methods for both approaches are discussed in detail in the Methods section.

## Results

**Figure 3:**
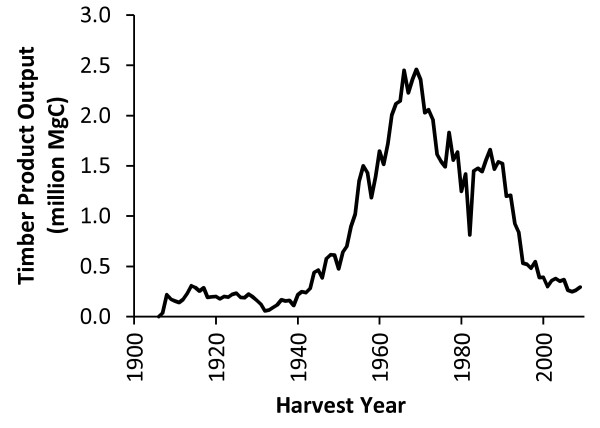
**Annual timber product output in the Northern Region, 1906 to 2010**. Annual timber product output in the Northern Region, 1906 to 2010 are based on data collected from USDA Forest Service Archives and Cut/Sold reports.

Between 1906 and 1943, the annual timber harvest in the Northern Region remained below 400,000 MgC yr^-1 ^(328.5 million cubic feet yr^-1^) and decreased during the Great Depression in the 1930's (Figure 3). After World War II, annual harvest levels increased steadily, with some volatility, to maximum harvest levels in the late 1960's and early 1970's. Growth in the annual harvest was particularly rapid between 1950 and 1956, when the annual harvest tripled from half a million MgC yr^-1 ^to 1.5 million MgC yr^-1 ^by 1956. At its peak in 1969, the annual timber harvest in the Northern Region exceeded 2.4 million MgC yr^-1^. Beginning in the mid-1970's, the annual harvest decreased steadily, with a brief increase in harvesting in the late 1970's followed by a particularly steep decrease during the economic recession of 1981-82. Between 1982 and 1987 the harvest level rose sharply, but then fell nearly every year from 1988 to 2002. Harvest levels since 2000 have been relatively stable between 200,000 to 400,000 MgC yr^-1^, which is similar to the harvest levels of the early twentieth century.

**Figure 4:**
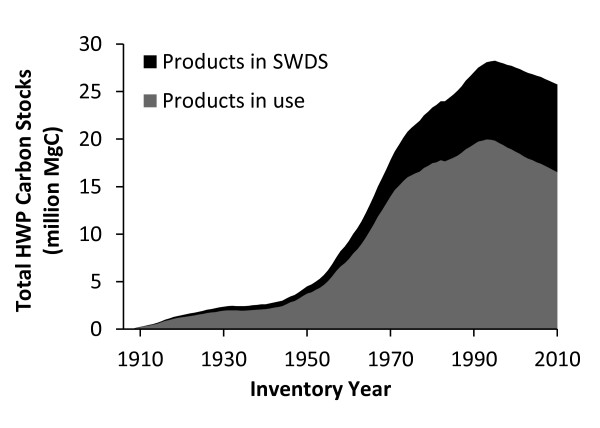
**Cumulative total carbon stored in HWP manufactured from Northern Region timber using the IPCC/EPA approach**. Cumulative total carbon stored in harvested wood products (HWP) manufactured from timber harvested from Northern Region National Forests using the IPCC/EPA production accounting approach. Carbon in HWP includes both products that are still in use and carbon stored at solid waste disposal sites (SWDS), including landfills and dumps.

All else being equal, higher harvest levels result in more carbon removed from the ecosystem pool and added to the HWP pool (Figure 2). Figure 4 shows the cumulative carbon in both the products in use and SWDS components of the HWP pool for the Northern Region using the production accounting approach. Using a format that matches the reporting for selected inventory years found in the most recent EPA report [2], Table 2 shows how the disposition of HWP carbon is broken into the four IPCC/EPA categories: emitted with energy capture, emitted without energy capture, products in use and products at SWDS. For each inventory year shown in the first column, the second column shows aggregate carbon emitted with energy capture (i.e. fuelwood), the third column shows aggregate carbon emitted through decay or combustion from SWDS, and the fourth and fifth columns show carbon stored in products in use and products at SWDS, respectively. The final column, the "Total remaining in the HWP pool," is the sum of products in use (column 4) and carbon at SWDS (column 5). It is important to understand that the estimate for each inventory year includes the portion of HWP carbon still in product in use and at SWDS for all previous harvest years back to 1906, in addition to carbon harvested in the inventory year. Some of the cumulative emissions from the burned and decayed HWP (Table 2, second and third columns) are theoretically taken out of the atmosphere by regrowth on harvested sites, but this effect is accounted for in the ecosystem carbon component of the IPCC/EPA approach (NEE), not in the HWP component (H and ΔC_R1_).

**Table 2:** Cumulative disposition of HWP carbon for selected years using the IPCC/EPA production accounting approach. This table shows the fate of all carbon removed from the ecosystem by harvesting.

Inventory year	Emitted with energy capture (MgC)	Emitted without energy capture (MgC)	Products in use (MgC)	SWDS (MgC)	TOTAL remaining in HWP Pool^a ^ (MgC)
1910	154,281	12,332	235,801	23,865	259,666
1920	957,662	196,962	1,271,481	219,922	1,491,403
1930	1,689,268	601,197	1,954,753	422,240	2,376,993
1940	2,125,441	1,118,607	2,116,591	511,967	2,628,558
1950	3,597,873	1,833,130	3,755,737	754,204	4,509,941
1960	7,561,338	3,672,609	7,394,180	1,894,409	9,288,589
1970	15,294,381	8,049,313	14,002,272	3,822,113	17,824,385
1980	22,072,575	13,859,456	17,464,432	5,855,782	23,320,214
1990	27,098,481	19,166,028	19,466,986	7,584,521	27,051,507
1995	29,034,443	21,725,124	19,855,947	8,396,262	28,252,209
2000	29,951,361	23,991,595	18,692,672	8,844,219	27,536,891
2005	30,634,194	25,950,852	17,589,954	9,084,182	26,674,136
2006	30,773,608	26,313,165	17,416,848	9,121,609	26,538,457
2007	30,884,369	26,664,054	17,186,329	9,151,940	26,338,269
2008	30,989,500	27,004,208	16,960,188	9,179,261	26,139,449
2009	31,112,619	27,333,955	16,743,418	9,204,640	25,948,058
2010	31,258,742	27,653,986	16,545,328	9,229,516	25,774,844

^a ^Sum of Products in use and SWDS.

**Figure 5:**
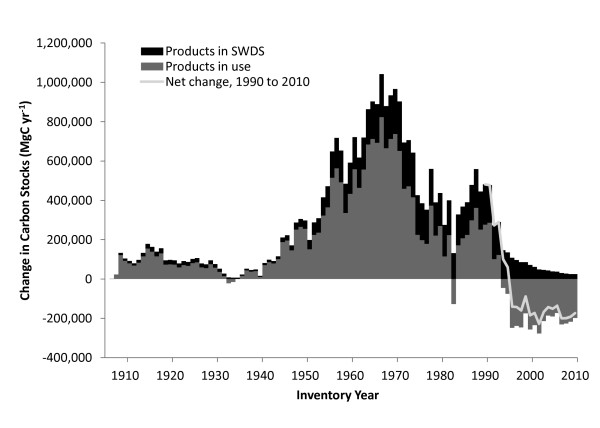
**The net change in carbon stocks in HWP from the previous year using the IPCC/EPA production accounting approach**. The net change in carbon stocks in HWP from the previous year using the IPCC/EPA production accounting approach. The net stock change is the sum of net change for SWDS (black bar) and products in use (gray bar). The total net change trend line from 1990 to 2010 shows a transition from net additions to carbon stocks in HWP to a period of net loss in carbon stocks in HWP.

The cumulative carbon stored in the Northern Region HWP pool peaked in 1995 at just over 28 million MgC. For reference, this is equivalent to 103 million MgCO_2_, the CO_2 _equivalent annual emissions from 20 million passenger vehicles. Since 1995, carbon stocks in the HWP pool for the Northern Region have been in decline (Figure 4). The 2010 HWP pool is estimated to be around 25.8 million MgC (Table 2). Figure 5 and Table 3 present the trend in terms of net annual change in HWP carbon stocks. Negative net annual change in HWP carbon stocks means the total carbon stored in the HWP pool in the inventory year is lower than in the previous year. A decline in the HWP pool results in a transition from a positive net annual change in carbon stocks to a negative net annual change in carbon stocks. In the mid-1960s, carbon stocks in HWP were growing by nearly one million MgC yr^-1^, with peak stock growth occurring during 1967 with the addition of 1,042,158 MgC yr^-1^. In the mid-1990's, the net change moves from positive to negative, and the HWP pool becomes a net source of atmospheric carbon. The year in the dataset with the largest net emissions from Northern Region HWP carbon pool was 2002, when a net of 228,241 MgC yr^-1 ^were emitted. These estimates relate only to HWP and do not quantify carbon fluxes in the ecosystem pool.

**Table 3:** Annual net change in HWP carbon stocks for selected years for harvests beginning in 1906 using the IPCC/EPA production accounting approach.

Inventory year	Stock change^a ^ (MgC yr^-1^)
1910	104,116
1920	97,021
1930	75,712
1940	16,051
1950	298,029
1960	591,785
1970	966,125
1980	437,628
1990	481,517
1995	59,764
2000	-184,812
2005	-151,437
2006	-135,679
2007	-200,187
2008	-198,821
2009	-191,391
2010	-173,214

^a ^Net annual change in carbon in products in use and SWDS

**Figure 6:**
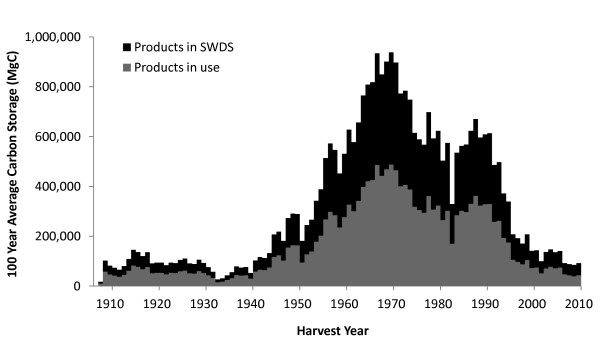
**Northern Region harvest 100-year average carbon HWP storage using the California Forest Project Protocol**. The 100-year average carbon storage in HWP for each year calculated for the Northern Region National Forest harvest using the California Forest Project Protocol. The 100-year average is calculated independently for each harvest year and considers only carbon harvested in that year.

The 100-year average calculated using the CFPP for the Northern Region, which is a projected average carbon stock over 100 years for harvest in a particular year, peaked in 1969 at 937,900 MgC (Figure 6). A declining trend in carbon storage in HWP since 1970 is also reflected by the 100-year averages (Figure 6, Table 4). In recent years, the 100-year average for the Northern Region has been between 84,000 and 150,000 MgC. Though the two estimation approaches differ in methods and calculations, they both show a clear and expected connection between timber harvest trends and carbon stored in the HWP pool.

**Table 4:** The 100-year average carbon stored in HWP for selected years using the California Forest Project Protocol.

Harvest year	Products in use^a ^ (MgC)	Landfills and dumps^b ^ (MgC)	Total (MgC)
1910	41,496	32,052	73,547
1920	52,862	40,832	93,694
1930	42,777	33,042	75,819
1940	57,768	44,621	102,389
1950	94,131	86,792	180,923
1960	326,709	301,238	627,947
1970	465,096	432,400	897,496
1980	264,336	238,992	503,328
1990	329,250	284,424	613,675
1995	105,200	102,218	207,418
2000	74,469	68,948	143,417
2005	73,794	66,384	140,177
2006	47,100	44,673	91,774
2007	42,728	45,027	87,755
2008	39,187	45,567	84,754
2009	43,541	48,377	91,917

^a ^The 100-year average carbon storage in products in use for the harvest year.

^b ^The 100-year average carbon storage in SWDS for the harvest year.

To quantify uncertainty, confidence intervals were estimated for both the IPCC/EPA HWP stock estimates and the CFPP 100-year average estimates using Monte Carlo simulation, representing 18 and 15 random variable distributions, respectively. Variable distributions were determined from publications and expert opinion. Table 5 shows the resulting confidence intervals for the IPCC/EPA estimates for selected years. For 1995, the year of peak carbon stocks for the Northern Region, the 90 percent confidence interval ranges from 20,723,740 MgC to 37,108,160 MgC, with a mean value of 28,272,940 MgC. This is equivalent to a -26.7 percent to +31.2 percent difference from the mean. Table 6 shows the resulting confidence intervals for the 100-year average for selected years. For 1970, the year with the highest 100-year average shown, the 90 percent confidence interval ranges from 563,303 to 1,336,731 MgC, with a mean value of 898,820 MgC. This is equivalent to a -37.3 percent to a +48.7 percent difference from the mean.

**Table 5:** Confidence intervals for cumulative carbon in HWP for selected years for harvests beginning in 1906. Means and confidence intervals were calculated using Monte Carlo simulation.

Inventory year	Simulation Mean (MgC)	90% Confidence interval	
		
		5% (MgC)	95% (MgC)
1910	258,847	151,997	383,534
1920	1,490,397	859,969	2,254,610
1930	2,380,130	1,348,945	3,726,131
1940	2,623,487	1,559,630	4,011,111
1950	4,508,105	2,756,788	6,754,915
1960	9,289,140	5,897,037	13,271,680
1970	17,825,210	11,508,690	25,465,070
1980	23,305,620	14,966,770	33,457,600
1990	27,036,780	19,354,590	35,924,610
1995	28,272,940	20,723,740	37,108,160
2000	27,510,220	20,779,040	35,696,240
2005	26,645,420	19,809,180	34,370,610
2006	26,538,740	19,630,590	34,125,580
2007	26,341,320	19,707,340	33,850,080
2008	26,128,290	19,561,330	33,672,940
2009	25,924,090	19,690,110	33,639,540
2010	25,753,020	19,546,530	33,052,480

**Table 6:** Confidence intervals for the 100-year average carbon stored in HWP for selected years using the California Forest Project Protocol. Means and confidence intervals were calculated using Monte Carlo simulation.

Inventory year	Simulation Mean (MgC)	90% Confidence interval	
		
		5% (MgC)	95% (MgC)
1910	73,654	41,230	117,343
1920	93,830	52,524	149,486
1930	75,929	42,504	120,967
1940	102,538	57,399	163,360
1950	181,181	113,537	270,498
1960	628,840	394,062	938,842
1970	898,820	563,303	1,336,731
1980	503,370	382,408	653,254
1990	613,729	463,510	803,920
1995	207,441	159,195	268,110
2000	143,439	108,755	186,617
2005	140,321	107,692	179,018
2006	91,865	70,445	116,970
2007	87,791	67,609	112,596
2008	84,825	66,493	106,480
2009	91,998	71,596	115,991

## Discussion

### HWP Carbon Estimates for the Northern Region

Although these results rely on numerous calculations, the time series of annual harvest volume (Figure 3) is at the root of the trends in carbon stocks and flux for the Northern Region HWP pool. Several recent publications help put these HWP carbon estimates in the context of the total forest carbon, including both ecosystem carbon and HWP carbon. By dividing our 2010 stock estimate of 25.8 teragrams of carbon (TgC) in HWP by the sum of this HWP estimate and Heath et al.'s [19] estimated 2010 Northern Region ecosystem carbon stock (25.8TgC in HWP plus 1,530 TgC total in ecosystem carbon), we estimate that the Northern Region HWP carbon stocks represent roughly 1.7 percent of total forest carbon storage associated with national forests in the Northern Region. At the national level, based on the EPA's estimate for 2010 total US HWP stock estimate of 2,449 TgC [2], the Northern Region HWP carbon stocks represented 1.1 percent of total US HWP carbon stocks.

Research efforts are under way to provide additional estimates of forest ecosystem flux in the West [13,15,19]. However, long-term data collection requirements will delay reporting until the National Forest Inventory and Analysis (FIA) program completes its second cycle of plot measurements. Although the third cycle has begun in some southern US states, it will be 2020 at the earliest in the Northern Region before second measurement data are available. Our calculations of HWP carbon flux will allow the Northern Region to reasonably account for carbon that was harvested between 1906 and 2010. Ideally, when changes in forest ecosystem carbon are quantified in subsequent research they can be linked with our HWP data.

### Applications of these approaches by forest managers

The availability of credible and practical methods for estimating this important carbon pool will allow resource managers and the public to develop a more complete understanding of the dynamics of HWP as a component of the forest carbon pool, and may allow the evaluation of the effect of alternative harvesting intensities on carbon stocks and fluxes. Furthermore, comparison of the two approaches is useful in evaluating the feasibility, utility, uncertainty, and limitations of alternative metrics and estimation methods that could be used to meet carbon monitoring objectives. Because the CFPP 100-year average is calculated for a discrete harvest year, data for previous harvest years is not needed to make current or future year estimates. This contrasts with the IPCC/EPA approach, which requires harvest information for many prior years to make an estimate of net change to the carbon stocks in the inventory year. The CFPP emphasis on harvest year calculations rather than annual changes in total carbon stocks makes the CFPP approach easier to apply when information on historical harvest and product disposition is lacking.

Similar to what we expect for other regions of the country, we had access to detailed recent information about wood harvest in agency "cut and sold" reports [20]. We were also fortunate to have archived historic harvest volume records. Although we made assumptions about the initiation of several primary product classes based on historical information, and we assumed consistent primary product distributions from the inception of processing capacity through the inventory year, in general we had a strong set of historical data to use in our calculations. As expected, records of the partitioning of the harvest to timber and primary product classes improved markedly as our records approached the present.

We recommend that all applications of the IPCC/EPA approach consider the quality of the data and adjust their uncertainty analysis accordingly - particularly with regards to the distributions of random variables. However, though carbon of older vintages may be associated with higher uncertainty, it is also likely to have a smaller impact on current stocks and fluxes than more recent harvests. For example, we estimated the importance of the early harvests by quantifying the portion in the current HWP pool that is attributable to carbon harvested prior to 1950. In 1950 the HWP carbon pool was 4.5 million MgC. By inventory year 2010, only 1.7 million MgC of the carbon harvested before 1950 remained in products in use and SWDS, which accounted for 6.6 percent of the total stocks of 25.8 million MgC in 2010. This small contribution to current stocks is a result of two factors. There was greater harvesting activity for the period after than before 1950. Also, following the passage of the Resource Conservation and Recovery Act of 1976 (RCRA) and after a short lag, a much larger portion of discarded HWP goes into modern landfills where it is subject to lower rates of decay than in aerobic dumps or disposal by open burning, which were the dominant disposal methods prior to RCRA.

Obtaining historical information may present a challenge for some regions and national forests. It may be particularly difficult to reconstruct harvest data prior to the mid-1940s, though regression of trends after the period might be appropriate for extrapolation to earlier periods. Alternatively, regions could base their carbon accounting on national level parameters, making the assumption that national-level numbers are adequate for regional and sub-regional analysis. If national level values represent the best available data, the IPCC/EPA method requires only harvest volume information from the user. Many regional and forest type-specific default dynamics and decay functions are supplied by national level work [3,9]. The simplicity associated with using national data in calculations may make the system functional and effective in meeting monitoring needs for forest managers both within and outside the National Forest System (NFS), regardless of data quality.

If time series data are not available or are very costly to procure, focusing on annual data may be more productive. The CFPP 100-year average is an example of an approach that does not require reconstructing the historical harvest. In general, the CFPP has ease of use superior to the IPCC/EPA production approach but does not provide the same detailed information about the HWP carbon pool. The CFPP approach does not estimate temporal trends in this pool whereas the IPCC/EPA approach can show both total stock and annual stock change. In addition, our results show that the effects of uncertainty appear to be higher for the 100-year average than for the IPCC/EPA estimates, for this case study, as measured as a percentage difference from the expected value. As with the IPCC/EPA calculations, appropriate regional and forest type-specific variables may be found in published sources [3,9,17].

The choice about which protocol should be applied could focus on the tradeoff between the simplicity of data collection and ease of calculations compared to a need to address both total stocks and flux. Also, managers may need to be consistent in all using one protocol or another in order to make results comparable across regions and easily aggregated in analysis done at larger scales. The more resource intensive methods of the IPCC/EPA estimates are worthwhile only if the additional detail is useful or if this reporting format is mandated.

We successfully applied the methods described by Skog [3] to estimate the uncertainty associated with our HWP carbon stock estimates (Table 6). However, it is unclear how the magnitude of this uncertainty would change, if at all, if the analysis were done on smaller management units (e.g. the individual national forest level). The change in uncertainty would, in large part, depend on assumptions made about the distributions of random variables used in the analysis. In some cases, a regional analysis may be sufficient to inform forest-level land management planning, forest management practices, and planning of long-term (programmatic) timber harvest levels and associated effects on carbon flux. A detailed sub-regional analysis may be needed where there are significant within-region differences in ecosystems and disturbance processes and harvest levels (e.g., western Washington compared to eastern Washington). In our case, Regional HWP carbon stocks can be meaningfully partitioned among the national forests in the Region based on harvest records. We are currently working to test these accounting methods, including uncertainty analysis, at smaller scales.

### HWP Carbon change estimates versus Life Cycle Assessment

There are well-developed methods of life cycle assessment (LCA) that account for all carbon emissions associated with manufactured products and that facilitate the comparison of different levels of consumption and substitution of wood products for alternative products [21]. Neither the IPCC/EPA nor the CFPP approach does this, which may be frustrating for some people interested in more than HWP stocks and stock change. For example, carbon emissions from fossil fuels used in transportation and manufacture of HWP are not deducted from the HWP pool. Similarly, though HWP emissions with energy capture are quantified in the IPCC/EPA approach, they are not assumed to substitute for an equivalent amount of fossil fuel carbon. Furthermore, these approaches do not incorporate carbon fluxes associated with product substitution, such as the substitution of HWP for metal or concrete (or vice versa) in building applications, and the associated land use changes that may ensue.

The IPCC/EPA and CFPP approaches instead focus on estimating physical stocks and fluxes of carbon in clearly defined forest carbon pools. This information can be used in an LCA, but does not address the same questions as an LCA. However, with some caution, these approaches can be used to estimate the effects of alternative past or future harvest levels on HWP carbon stocks and fluxes. For example, a hypothetical time series of harvest volumes can be used to predict what future product storage and emissions would look like under specific alternative forest management and harvest scenarios. But this is not an effective proxy for a consequential LCA, which might include harvesting, transportation and processing emissions, as well as product substitutions, and other trade components not included in the two approaches used here. For sub-national carbon accounting, IPCC/EPA and CFPP approaches have several benefits over LCA. They are relatively easy to apply and congruent with US national carbon accounting standards, which is particularly important in developing tools that can be used by USFS managers to meet carbon monitoring goals.

Together with accounting and modeling methods that quantify ecosystem forest carbon, the approaches used in this study provide a powerful tool to monitor carbon stocks, stock change, and 100-year averages, as well as the ability to assess the possible outcomes of management actions intended to reduce the vulnerability of forest resources to climate change.

## Conclusions

HWP is an important carbon pool that should be considered in decision making associated with carbon monitoring and climate change adaptation and mitigation. In this analysis, we have found that when harvest and wood product production data exist, national and state level accounting protocols can be applied effectively at finer scales, such as regional, national forest, or individual landowner scales. The Northern Region HWP pool is now in a period of negative net annual stock change because the decay of products harvested between 1906 and 2010 exceeds additions of carbon to the HWP pool through harvest. However, total forest carbon is a function of both HWP and ecosystem carbon, which may have increased over the study period. We also demonstrate methods for quantifying uncertainty to describe the confidence we have in estimates of their carbon storage metrics. However, there are clear tradeoffs between alternative approaches to estimating HWP carbon stocks. The CFPP 100-year average uses harvest year data and is easier to apply, but it does not provide information about total carbon stocks or annual stock change. In comparison, the IPCC/EPA production accounting approach is more data intensive because it includes past harvest and product disposition data for each inventory year, but it provides estimates of total stocks and stock change, which makes it congruent with national accounting and reporting protocols. The IPCC/EPA approach could be used to predict changes to the HWP component of the forest sector carbon pool resulting from planned or potential change in the amount of wood harvested. We believe further research is necessary to help policy makers and managers better understand the implications of alternative forest management strategies on forest carbon stocks and stock change. An integrated approach might include consequential LCA that evaluates changes in harvest activity on carbon emissions including all sources of emissions and product substitutions.

## Methods

### Data Sources

Northern Region harvesting activity since 1980 has been reported in detailed cut and sold reports. These reports include the value and volume of timber sold and harvested in the region, which are reported by both fiscal and calendar year [22]. In addition, the total harvest is partitioned by sale value, timber product (e.g. softwood sawlogs), tree species, and by each national forest within the region. Beginning in 2001, volumes have been reported in both thousand board feet (mbf) and hundred cubic feet (ccf). Between 1980 and 2000, volumes were reported in mbf only. For these years, regional conversion factors for specific timber products were used to convert volumes from mbf to ccf (Table 7).

**Table 7:** Conversion factors used in this analysis.

Conversion	Units
2.2	ccf per mbf, timber harvest
1.75 to 2.56	ccf per mbf, timber products
33 to 42	lbs per cubic foot, primary products
2204.6	lbs per Mg
0.95 to 1.0	Mg wood fiber per Mg product
0.5	Mg carbon per dry Mg wood fiber
0.711 to 0.919	MgC per ccf, primary products

Records for annual harvest prior to 1980 are more difficult to obtain. Paper records are available in the Northern Region archives for fiscal years 1946 to 1979, but these do not report the harvest by timber product classes and vary in their reporting for individual national forests. The forestland included in the Northern Region and the administrative designations of specific forests has changed over time. Whenever possible, we used forest-specific data to standardize harvest totals from 1946 to 1979 to the modern boundary of the Northern Region. For example, the harvest for Colville National Forest, which was transferred to the Pacific Northwest Region in 1975, was removed from the harvest totals reported for the Northern Region from 1946 through 1974.

Documents from the archives also report the annual harvest in mbf for fiscal years 1906 to 1920, calendar years 1921 to 1936, and fiscal years 1937 to 1945. In these records, the harvest is divided into three administrative designations for years 1914 to 1945, but data for individual forests and timber products are not reported for the period 1906 to 1945. Again, we adjusted annual harvest data in an attempt to standardize the harvest to the modern boundaries of the Northern Region. For years prior to 1946, the annual harvest reported for the Northern Region was reduced by 5.3 percent, which is the average proportion of the Northern Region annual harvest attributable to Colville National Forest from 1946 to 1971. The proportion of the harvest attributable to Colville National Forest stayed relatively constant over this period, ranging from a low of 2.4 percent in 1946 to a high of 7.2 percent in 1965.

For the period 1906 through 1979, annual harvest totals for the Northern Region reported in mbf were converted to ccf using a conversion factor of 2.2 ccf per mbf (Table 7). Harvest records during this period do not partition the harvest among different timber product classes. To split the harvest among the different product classes, we first worked with our local Timber Product Output specialists [22] to estimate the dates when various processing operations commenced in our study area. Next, we applied the average annual proportion of the harvest represented by each timber product class from 1980 through 2009 to the annual harvest for each year 1906 through 1979 (Table 8). By standardizing boundaries and units and partitioning the harvest among different timber product classes, we created a continuous dataset spanning 1906 through 2009 that meets the criteria for estimation established by the IPCC [8].

**Table 8:** The average annual proportion of the Northern Region harvests distributed to timber product classes between 1980 and 2009.

Product class	Mean (%)	Std. error
Sawtimber, softwood	78.7	1.70
Pulpwood, softwood	6.9	0.78
Fuelwood, softwood	8.6	1.11
Non-saw, softwood	2.0	0.82
Other products	3.8	0.41

### Computational Methods

**Figure 7:**
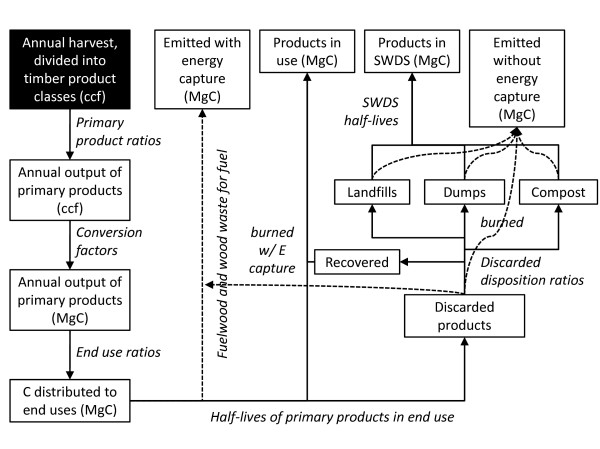
**Schematic for calculations to quantify HWP storage and emissions**. Schematic showing the flow of calculations to quantify HWP products in use, products in SWDS, emissions with energy capture, and emissions without energy capture using the IPCC/EPA approach.

Figure 7 provides a flow chart of the computational methods used to calculate annual stock changes and emissions from HWP for the IPCC/EPA production accounting approach. This approach does not apply simple storage ratios to the harvest; it follows carbon through the product life cycle from harvest to timber products to primary products to end use to disposal, applying appropriate ratios and half-lives at each stage. Annual volumes of output for specific timber product classes (e.g. softwood sawlogs) are distributed to specific primary products (e.g. softwood lumber, softwood plywood, etc.) using average primary product ratios for the Rocky Mountain Region from Smith et al. [9]. Primary product outputs are converted from their reporting units to MgC using standard conversion factors for primary products [9] (Table 7).

The recalcitrance of carbon in harvested wood products is highly dependent upon the end use of those products. For example, carbon in lumber used in new single family home construction has a longer duration of use than carbon in lumber used for shipping containers, which is released into the atmosphere more quickly through combustion and decay. For years 1950 through 2006, annual primary product output was distributed to specific end uses according to annual wood product consumption estimates in McKeever [23]. Estimates for 1950 were used for 1906 through 1949 and estimates for 2006 were used for 2007 through 2009. We acknowledge that this is not ideal, but no other data are available for these periods. For each end use and vintage year, the amount of carbon remaining in use at each inventory year is calculated based on the product half-life and the number of years that have passed between the year of harvest and the inventory year. The half-life value expresses the decay rate at which carbon in the products in use category passes into the discarded category. The carbon remaining in HWP in a given inventory year is calculated for each vintage year end use based on a standard decay formula:

$${\text{N}}_{\text{t}}=\;{\text{N}}_{0}\text{exp}\left(-t\text{ln}\left(\text{2}\right)∕{\text{t}}_{\text{1}∕\text{2}}\right)$$

where N_t _is the amount of carbon remaining in use in inventory year t, N_0 _is the amount of carbon in the end use category in the vintage year of harvest, t is the number of years since harvest, t_1/2 _is the half-life of carbon in that end use, and exp is notation for the exponential function. In our calculations, the starting amount (N_0_, at n = 0) is adjusted downward by 8 percent to reflect a loss when placed in use, which is assumed to enter the discarded carbon category. This loss in use accounts for waste when primary products (e.g. softwood lumber) are put into specific end uses (e.g. new single family residential housing). Fuelwood products are assumed to have full emissions with energy capture in the year they were produced (Table 2, Figure 7).

For carbon of a particular vintage in a given inventory year, the balance of carbon in HWP that is not in use and not emitted with energy capture is assumed to be in the discarded products category (Figure 7). Carbon in the discarded products category is partitioned into five disposition categories: burned, recovered, composted, landfills and dumps. The proportion of discarded products that ends up in each of these five categories is different for paper and solid wood products, and has changed over time. For example, prior to 1970 wood and paper waste was generally discarded to dumps, where it was subject to higher rates of decay than in modern landfills. Since then, the proportion of discarded wood going to dumps has dropped to below 2 percent, while the proportion going to landfills has risen to 67 percent, with the remainder going to the other disposition categories. Similarly, composting and recovery (i.e. recycling and reuse) have become a more prominent part of waste management systems. In 2004, approximately 50 percent of paper waste was recovered, compared to 17 percent in 1960. The disposition of carbon in paper and solid wood products to these categories is based on percentages in Skog [3].

Carbon from burned and composted discarded products is assumed to be emitted without energy capture. Carbon in the recovered category reenters the products in use category. Carbon in products discarded to landfills and dumps are subject to decay determined by their respective half-lives. The half-life value for discarded products in dumps and landfills expresses the decay rate at which carbon in these categories is emitted to the atmosphere. However, only a fraction of discarded products in landfills is considered to be subject to decay. Seventy-seven percent of solid wood carbon and 44 percent of paper carbon in landfills is identified as fixed carbon, not subject to decay [3]. For a given vintage year, the carbon remaining in SWDS in a given inventory year is the sum of fixed carbon and the carbon remaining after decay. We do not account for the difference between methane and carbon dioxide emissions from landfills in terms of CO_2 _equivalents, nor do we account for methane remediation that includes combustion and subsequent emissions with energy capture. All landfill and dump emissions are considered emissions without energy capture.

IPCC/EPA methods were used to calculate HWP carbon stocks and stock change for inventory years 1906 through 2010. In addition, we present the 100-year average carbon storage for products in use, products in SWDS, and all HWP using the CFPP. In this approach, averages for each harvest year are determined based on storage factors for primary wood product classes as described previously. The amount of carbon delivered from a harvest to mills is determined by applying conversion factors by wood type. However, not all of the carbon that is delivered to the mill ends up in HWP. The amount of delivered carbon that ends up in HWP is determined by mill efficiency factors of 67.5 percent for softwood and 56.8 percent for hardwoods, with the balance of carbon assumed to be immediately emitted to the atmosphere. These mill efficiency factors determine the total carbon transferred into HWP in the year of harvest. For each harvest year, the average carbon stored over 100 years in wood products harvested in that year is calculated based on 100-year average storage factors applied to the HWP carbon in different wood product classes, with different factors applied for the in use and in landfills HWP carbon categories [17]. The total average carbon stored in HWP over 100 years for each harvest year is calculated as the sum of the in-use and landfill averages, and only includes HWP carbon harvested in that harvest year.

In our calculations the carbon in HWP to which the CFPP storage factors are applied is based on conversion, residue production, and product recovery factors incorporated into the production accounting approach, not on the conversion factors and generalized mill efficiency factors included in the CFPP. In other words, the results presented here apply the CFPP protocol to the same HWP carbon pool used in the production accounting approach, which is shown in Figure 3. Using the same pool removes variability that would be attributable to applying different conversion factors and allows a more clear comparison of the two accounting systems.

### Uncertainty and Limitations

Interpretation of the results should be made in light of some constraints. Though we attempted to normalize annual harvests to the modern boundary of the Northern Region using forest-specific harvest data, in actuality the annual harvest is from a land base that is somewhat variable over time. The USFS has commonly engaged in land exchanges, divestments and acquisitions in the Northern Region since 1906, which means that the system boundary for this analysis is not consistent. In addition, conversion factors, the distribution of timber products to primary products, and the distribution of primary products to end uses have changed over time. Though we have used annual data whenever possible, there is some uncertainty associated with applying averages to annual harvests in the early years of this harvest series.

Uncertainty is quantified using the methods described by Skog [3]. We identified the most critical sources of uncertainty in our analysis (Table 9), developed probability distributions for each, and carried out Monte Carlo simulations to determine the effect of uncertainty in these variables on estimates of HWP stocks and 100-year average. The 18 random variable distributions in Table 9 represent four major sources of uncertainty: conversion factors, reported harvest, product distribution variables, and product decay parameters. Because we apply different distributions to different time periods for some variables, the 18 distributions cover 12 different variables. Multiple time-delineated distributions are used for timber products conversion factors, reported harvest, proportion of roundwood going to softwood sawtimber, and proportion of softwood sawtimber going to lumber manufacturing, with time periods separated at benchmark years related to data quality. Analysis for the IPCC/EPA approach uses all 18 distributions, but analysis for the CFPP approach uses 15 because only one of the product decay variables (product half-lives/storage factor) is used in CFPP calculations.

**Table 9:** Sources of uncertainty and associated data.

Source of Uncertainty	Specific Factor	Years	Relevant products	90% CI
Conversion factors	mbf:ccf	1906-1979	Timber products	± 30%
	mbf:ccf	1980-2009	Timber products	± 15%
	ccf:MgC	1906-2009	Primary products	± 5%
Reported harvest	Harvest in mbf	1906-1945	Timber products	± 30%
	Harvest in mbf	1946-1979	Timber products	± 20%
	Harvest in mbf or ccf	1980-2009	Timber products	± 15%
Product distribution	Roundwood to softwood sawtimber	1906-1979	Timber products	± 30%
	Roundwood to softwood sawtimber	1980-2009	Timber products	± 15%
	Softwood sawtimber to lumber	1906-1949	Timber products	± 30%
	Softwood sawtimber to lumber	1950-1979	Timber products	± 20%
	Softwood sawtimber to lumber	1980-2009	Timber products	± 15%
	Lumber going to new housing	1906-2009	Primary products	± 15%
	Panels going to new housing	1906-2009	Primary products	± 15%
	Residues going to pulp	1906-2009	Primary products	± 15%
Product decay	Product half-life or storage factor	1906-2009	All end-use	± 15%
	Fraction of discards to landfills	1906-2009	Discarded	± 15%
	Landfill decay limits	1906-2009	Landfilled	± 15%
	Landfill half-life	1906-2009	Landfilled	± 15%

The probability distributions of these random variables were developed based on estimates in Skog [3] and on professional judgment, and are assumed to be triangular and symmetric. The distributions are also assumed to be independent of one another. However, in the simulation, a correlation coefficient of 0.5 was applied to reported harvest for the three time periods to quantify the assumption that if the harvest was systematically overestimated or underestimated in one period, it was likely in error in the same direction in the other two periods (Table 9). A similar approach was used for product half-lives in the product decay category to quantify the assumption that if half-lives for one category were underestimated or overestimated, the other categories were likely in error in the same direction. In general, uncertainty was assumed to be greater farther back in time. For example, reported harvest is divided into three time periods based on data quality, with uncertainty in the reported harvest increasing from ± 15 percent for the period from 1980 to 2009, to ± 20 percent for the period from 1946 to 1979, to ± 30 percent for the period from 1906 to 1945.

The effect of uncertainty on the HWP stocks and 100-year average was evaluated using Monte Carlo simulation in @Risk software version 5.7 [24]. The simulation means and 90 percent confidence intervals shown in Tables 5 and 6 are the results of 2,200 iterations with Latin hypercube sampling, which was determined to be the average number of draws needed to reach a stable standard deviation for the estimate of HWP stocks.

## Competing interests

The authors declare that they have no competing interests.

## Authors' contributions

KDS built a team to carry out the project, entered historical harvest data, helped with estimation procedures and data management decisions, and compiled and edited the manuscript. NMA designed the spreadsheets to perform calculations, entered most of the data, prepared results, wrote the methods and results sections of the manuscript, and edited the manuscript. KES made substantive intellectual contributions by first steering the team to the existing EPA/IPCC and CFPP protocols, then by applying his and other's recently published work to this particular system, by directing the uncertainty analysis, and by reviewing manuscript drafts. SPH contributed intellectually by sharing recent ecosystem carbon estimation approaches, reviewing initial system design, and outlining sections of the manuscript. DRL and GJ contributed to study design, helped validate and adjust historically-based estimates, and participated in preparing the manuscript. JFM provided important information regarding the need for the project, drafted the background section of the manuscript and facilitated internal draft reviews. All authors read and approved the final manuscript.
